# Length of efficacy and effect of implant location in adult tom cats treated with a 9.4 mg deslorelin subcutaneous implant

**DOI:** 10.1177/1098612X18788157

**Published:** 2018-07-30

**Authors:** Stefano Romagnoli, Anna Baldan, Silvia Ferro, Camilla Righetti, Ludovico Scenna, Gianfranco Gabai, Tamara Badon, Christelle Fontaine, Antonio Mollo, Calogero Stelletta, Chiara Milani

**Affiliations:** 1 Department of Animal Medicine, Production and Health, University of Padova, Padova, Italy; 2 Private Practitioner; 3 Department of Comparative Biomedicine and Food Science, University of Padova, Padova, Italy; 4 Virbac, Carros, France

**Keywords:** Tom cat, deslorelin, reversible sterility, reproduction control, fertility

## Abstract

**Objectives:**

The objective of this study was to assess duration of efficacy, side effects and return to fertility following use of the 9.4 mg deslorelin implant (Suprelorin 12; Virbac) in cats, and test whether efficacy and duration of action are influenced by implantation site (interscapular vs periumbilical).

**Methods:**

Sixteen healthy adult tom cats were checked with (1) reproductive examination, (2) gonadotropin-releasing hormone stimulation test and (3) semen collection until achievement of sterility, then with (1) and (2) only at 2, 4, 6 and 12 months, and every 6 months thereafter until treatment effect disappeared.

**Results:**

Serum testosterone reached basal levels by 7 days post-treatment. Semen quality improved initially then started to worsen by 1 month post-treatment and after 70 days post-treatment all cats were sterile. Early in the third month post-treatment there was a significant decrease in testicular volume and penile spikes. Testicular histology was normal upon neutering performed after resumption of fertility. No injection site lesions or treatment-related side effects were observed. There was no difference between periumbilical and interscapular placement for all criteria, but there was a trend for the decrease in testicular volume to last longer and for the regression of penile spikes to start sooner after interscapular administration. One of 16 cats did not respond to treatment. Six cats were lost at variable times during the study while fully responding to treatment. In the cats that completed the study, normal fertility was regained after 805 days, on average, but with a variable duration of effect from 750–850 days.

**Conclusions and relevance:**

Treatment with a 9.4 mg deslorelin implant in male cats was effective for a period of 750–850 days, which is 1.5–2 times longer than the effect of the 4.7 mg deslorelin implant. Fertility (based on serum testosterone production and the presence of penile spikes) was regained at the end of the study. Placing implants in the intrascapular vs periumbilical location did not affect duration of suppression of testosterone production. The interscapular location may be characterised by a better efficacy, although further studies are needed to clarify this issue.

## Introduction

Deslorelin is a gonadotropin-releasing hormone (GnRH) agonist that has become commercially available as a reproduction-control drug for use in adult male dogs. Its prolonged action, causing pituitary downregulation, allows for a sustained block of gonadal action that is effective in causing temporary sterility in male dogs.^1–8^ The achievement of a temporary blockage of fertility is becoming a common request in small animal practice, and particularly so in feline practice as cat breeders may decide to alternate the use of their tom cats. Also, pet owners are becoming increasingly concerned about the use of irreversible techniques and may request the option of reversible pharmacological castration in order to delay the choice of surgery.

A 4.7 mg deslorelin implant currently marketed for use in male dogs is increasingly being used off-label in both male and female cats.^9–12^ The extra-label use of deslorelin in male cats is becoming popular in feline practice owing to its similar safety profile and prolonged effectiveness when compared with male dogs.^9–15^ Deslorelin implants are commonly placed subcutaneously in the interscapular area. A periumbilical placement is regarded as more practical owing to the skin being thinner and often devoid of fat, which makes the implant visible for a long time. However, to the best of our knowledge, there is no information in cats on efficacy and duration of action of a deslorelin implant placed in the periumbilical as opposed to the interscapular area.

Recently, a 9.4 mg version of the deslorelin implant was introduced to the European market with the same indications (fertility control in dogs) as the 4.7 mg compound and twice the duration of action. The objectives of this study were to: (1) assess duration of efficacy, side effects and return to fertility in adult male cats treated with a 9.4 mg deslorelin implant; and (2) test whether efficacy and duration of action of such implants in tom cats are the same, regardless of the site of implantation.

## Materials and methods

The University of Padova Ethics Committee approved the study (Project number 64 bis/2011). Sixteen privately owned adult tom cats of various breeds (14 Europeans, one Birman and one Turkish Angora), aged 5 months to 5 years and weighing 3.0–5.8 kg were recruited with the owners’ consent. Health conditions were assessed at the beginning of the study through a general clinical examination and blood collection for haematology (ADVIA 120 Hematology System; Siemens) and biochemistry (BT 1500; Biotecnica). Weight was checked with an electronic scale (VT 2 model; Wunder). The post-pubertal state of the cats was assessed through reproductive history and a clinical reproductive examination, which included measurement of testicular size and volume, observation of penile spikes on the penile mucosa, and live and motile spermatozoa on semen collection. Relevant clinical observations (testicular volume measurements, observation of penile spikes) were made independently by two operators.

All cats were administered a 9.4 mg deslorelin implant (Suprelorin 12; Virbac). Deslorelin implants were inserted on alternate cats in two different subcutaneous locations: interscapular (in between the dorsal aspects of the shoulder blades) and periumbilical (within the subcutaneous tissue about 1.0 cm cranial to the umbilical scar). Skin disinfection with iodine solution was performed for both placements, and hair trichotomy of an area approximately 7 × 3 cm was performed for periumbilical placement. No sedation was performed and no skin stitches were placed following implant insertion

In order to monitor efficacy and length of treatment, cats were periodically checked for: (1) reproductive behaviour; (2) body weight; (3) observation of penile spikes; (4) measurement of testicular volume; (5) blood collection for serum testosterone assay; and (6) semen collection.

Semen collection was performed, as previously described, by inserting a urinary catheter 9 cm into the urethra 10 mins after sedation obtained with intramuscular injection of 100 μg/kg medetomidine, every 2 weeks until complete sterility was reached, and was repeated at the end of the study.^16^ Sterility was defined as either sperm motility <20%, normal morphology <20% or total number ⩽0.5 × 10^6^ of spermatozoa.^17^ Semen collection was performed at the end of the study in order to confirm resumption of fertility. From the twelfth month post-treatment onwards cat owners were consulted by telephone on a monthly basis and as soon as they reported a change in reproductive behaviour of their animals the tom cats were re-checked. Return to fertility was based on the presence of normal testosterone concentrations (⩾1.0 ng/ml)^18,19^ and/or normal or re-growing penile spikes.

Tom cats were orchiectomised following return to fertility (unless the owners preferred to maintain their cats intact) and testicular histology was carried out in order to confirm return to normal reproductive function. After fixation of the testicles in Bouin’s solution (Sigma-Aldrich) 4 μm serial sections of each testicle stained with haematoxylin and eosin were examined with an optical microscope (Olympus BX40) to evaluate testicular (assessing presence of spermatogonia, spermatids, mature spermatozoa, Sertoli and Leydig cells) and epididymal (epithelial changes and presence of spermatozoa) parenchyma.

Serum testosterone assay was measured on samples collected 1 h after intravenous (IV) or intramuscular (IM) injection of 50 μg/cat gonadorelin (Fertagyl; Intervet)^20^ at 0 (day of treatment), 7 and 15 days post-treatment, then every 2 weeks until sterility was reached, then at 4 and 6 months post-treatment, and thereafter every 6 months. Sera were obtained from fasted animals by jugular venepuncture using dry test tubes with clot activator (FL Medical) centrifuged at 1750 *g* for 10 mins and the serum separated and aliquoted. Serum testosterone was assayed using chemiluminescence (Immulite 1000; Medical System). Validation of the chemiluminescent method for feline testosterone assay was done by means of linearity and recovery tests. For linearity, serum aliquots from 13 male cats in this study were used. Following initial assay of serum testosterone, high (T-High, 10 ng/ml) and low (T-Low, <0.1 ng/ml) testosterone pools were made: the T-High pool was aliquoted and diluted with the T-Low pool in order to obtain the following dilutions: 1:2, 1:4, 1:8, 1:16, 1:32 and 1:264. For recovery test, T-Low was spiked with synthetic testosterone (Sigma-Aldrich) diluted in ethanol, in order to obtain the final expected concentrations of 0.0, 0.7, 1.40, 2.80 and 5.60 ng/ml.

Testicular volume was calculated using the following formula:^21^ L × W × H × 0.5236. Length (L), width (W) and height (H) were measured using a calliper.

**Figure 1:**
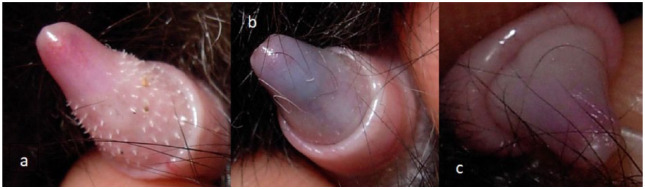
Scoring of penile spike appearance in tom cats treated with 9.4 mg deslorelin implants. (a) Presence of penile spikes = score 1. (b) Regressing/re-growing penile spikes = score 2. (c) Absence of penile spikes = score 3

Penile spikes were checked by manually protruding the prepuce and classifying them as present (score = 1), regressing/re-growing (score = 2) and absent (score = 3) (Figure 1).

### Statistical analysis

Duration of treatment effect was investigated with a two-way ANOVA (GLM procedure, SIGMASTAT 2.03) using time post-treatment (PT) (0 = day of implant, 1 = 1–60 days PT, 2 = 61–180 days PT, 3 = 181–360 days PT, 4 = 361–700 days PT, 5 = >700 days PT) and implant location (interscapular vs periumbilical) as independent variables, and weight, testicular volume, serum testosterone and penile spikes as dependent variables. Differences in duration of effect and implant location on semen quality were investigated with a one-way ANOVA using either time PT or implant location (interscapular vs periumbilical implant placement) as the independent variables and progressive motility (%), morphology and spermatozoa concentration as dependent variables. Pearson’s correlation was calculated for all parameters. Significance was set as *P* <0.05. Differences on serum testosterone concentration were investigated with a one-way ANOVA, considering all the cats of the study, irrespective of implant location and using time PT as the independent variable, and weight, testicular volume, serum testosterone and penile spikes as dependent variables.

## Results

All cats were in good general health at the beginning of the study with normal haematological and biochemical parameters. Deslorelin implants were placed in the interscapular and periumbilical areas in 10 and six cats, respectively. The difference in group allocation was a result of two clients objecting to periumbilical placement. The length of monitoring periods for each single animal varied as five cats were lost early in the study: three cats disappeared 40 days PT (cats 3, 5 and 16) and two 8 months PT (cats 4 and 8). Extended monitoring could be carried out on 11/16 cats (six from the interscapular and five from the umbilical group) (Tables 1–3). Two of these 11 cats (cats 12 and 14) were neutered at 11 and 12 months PT, respectively, because of early resumption of roaming and mounting behaviour, despite basal (<0.1 ng/ml) serum testosterone concentrations and absence of penile spikes. Cat 1 was hit by a car and died 25 months PT. Not all periodic checks could be made owing to lack of owner compliance; as a consequence, for example, serum testosterone in the first week PT could be measured only on four cats. Also, not all clinical observations could be made independently by two different operators.

**Table 1:** Weight, post-gonadotropin-releasing hormone serum testosterone concentrations, testicular size, presence of penile spikes, semen quality and reproductive behaviour at differing numbers of days post-treatment within each of the time periods for six cats (cats 2, 3, 6, 9, 10, 12) treated with a 9.4 mg deslorelin implant in the periumbilical area

Time period	Cat	Days PT	Weight (kg)	Testosterone (ng/ml)	Right testicle (cm^3^)	Left testicle (cm^3^)	Penile spikes*	Semen quality	Reproductive behaviour
0	2	0	5.8	12.73	0.980179	0.980179	1		
	3	0	4.6	9.66	0.551351	0.551351	1		
	6	0	4.7	14	1.238838	1.238838	1		
	9	0	3.6		0.62832	0.62832	1		
	10	0	3.2	2.81	0.335104	0.335104	1		
	12	0	4		0.7854	0.7854	1		
1	2	9	5.7		0.980179	0.980179	1		
	12	14	4.1	0.543	0.7854	0.7854	1		
	6	15	4.4		1.23884	1.238838	1		
	10	19	3	0.1	0.3351	0.3351	1		
	9	20	3.6	0.1	0.62832	0.62832	1		
	9	33	3.8	0.1	0.424116	0.424116	2		
	10	35	3.3	0.1	0.335104	0.335104	2		
	3	40	4.4	0.1	0.551351	0.551351	2		
	6	40	4.6	0.513	0.886978	0.886978	2		
	12	41	4.2		0.68068	0.68068	1		
	9	48	3.8	0.1	0.30159	0.30159	3		
	10	49	3.2	0.1	0.335104	0.335104	3		
2	9	62	3.8	0.1	0.30159	0.30159	3		
	10	63	3	0.1	0.335104	0.230908	3		
	12	64	4.1	0.1	0.466528	0.368614	2		
	2	66	5.2	0.1	0.424116	0.424116	2		
	6	85	5		0.62832	0.62832	3		
	10	99	3.3	0.1	0.3351	0.23091	3		
	9	104	3.9	0.1	0.30159	0.30159	3		
	6	111	4.9	0.27	0.62832	0.62832	3		
	2	114	5.2	0.1	0.301594	0.301594	3		
	12	124	4.6	0.1	0.230908	0.169646	3		
	9	160	4.15		0.205251	0.205251	3		
	10	161	4.1		0.150797	0.09163	3		
	2	166	5.2	0.1	0.30159	0.30159	3		
	6	179	5.1		0.62832	0.62832	3		
3	2	215	4.6		0.30159	0.30159	3		
	10	238	3.6		0.1319	0.07854	3		
	9	244	4.2		0.205251	0.205251	3		
	12	245	6.7	0.1	0.368614	0.368614	3		Marking, mounting
	6	257	5.3		0.424116	0.424116	3		
	2	292	5.2		0.230908	0.230908	3		
	10	344		0.164			3		
4	10	470		0.25	0.4	0.4	3		
	9	520	4		0.47	0.47	3		
	2	530	5.2	15.2	0.37	0.37	3		
	6	600		0.1	0.47	0.47	3		
	10	600		0.1			3		Mounting
	10	690					1		Marking, masturbation
	6	700					1		Calling females
5	9	714	3.8		0.57	0.57	3		
	10	750		15.22	0.5	0.5	1	Fertile (70% motility)	Mounting, marking, masturbation
	2	783	5	15.1	0.52	0.52	1		Roaming
	6	851		11.42			1	Fertile (80% motility)	Three kittens
	9	895	3.8		0.57	0.57	1		
	2	943	5	16	0.52	0.52	1	Fertile (230 × 10^6^ sperm, 80% motility)	Calling females

Time periods: 0 = day of implant; 1 = 1–60 days post-treatment (PT); 2 = 61–180 days PT; 3 = 181–360 days PT; 4 = 361–700 days PT; 5 = >700 days PT

* 1 = present; 2 = regressing/re-growing; 3 = absent

**Table 2:** Weight, post-gonadotropin-releasing hormone serum testosterone concentrations, testicular size, presence of penile spikes, semen quality and reproductive behaviour at differing numbers of days post-treatment within each of the time periods for 10 cats (cats 1, 4, 5, 7, 8, 11, 13, 14, 15, 16) treated with a 9.4 mg deslorelin implant in the interscapular area

Time period	Cat	Days PT	Weight (kg)	Testosterone (ng/ml)	Right testicle (cm^3^)	Left testicle (cm^3^)	Penile spikes*	Semen quality	Reproductive behaviour
0	1	0	3	9.81	0.62832	0.62832	1		
	4	0	4.7	10.88	0.68068	0.68068	1		
	5	0	4.1	7.03	1.64201	1.64201	1		
	7	0	5.5	16	2.00277	2.00277	1		
	8	0	3.5	6.76	1.055578	1.055578	1		
	11	0	4.3	37.24	1.130976	1.130976	1		
	13	0	2.7	10.08	0.42	0.42	1		
	14	0	3.7	1.71	0.6	0.6	1		
	15	0	3.2	2.41	0.5	0.5	1		
	16	0	3.6	8	0.47	0.47	1		

Time periods: 0 = day of implant; 1 = 1—60 days post-treatment (PT); 2 = 61—180 days PT; 3 = 181—360 days PT; 4 = 361—700 days PT; 5 = >700 days PT

* 1 = present; 2 = regressing/re-growing; 3 = absent

**Table 3:** Implant location, time period, body weight, left and right testicular volume, presence of penile spikes and post-gonadotropin-releasing hormone serum testosterone concentration in 16 male cats implanted with a 9.4 mg deslorelin implant

Implant location	Time period (days)	Body weight (kg)	Left testicular volume	Right testicular volume	Penile spikes*	Serum testosterone
Interscapular	0	3.956 ± 0.276 (n = 9)	0.968 ± 0.09 (n = 10)	0.968 ± 0.09 (n = 10)	1 ± 0.214 (n = 9)	11.093 ± 2.199 (n = 9)
	1 (1–60)	4.143 ± 0.214 (n = 15)	0.646 ± 0.07 (n = 15)	0.646 ± 0.07 (n = 15)	1.8 ± 0.166^†^ (n = 15)	0.188 ± 1.135^†^ (n = 15)
	2 (61–180)	4.550 ± 0.221 (n = 14)	0.41 ± 0.07^†^ (n = 14)	0.41 ± 0.07^†^ (n = 14)	2.571 ± 0.172^†^ (n = 14)	0.1 ± 1.555^†^ (n = 8)
	3 (181–360)	4.380 ± 0.370 (n = 5)	0.319 ± 0.114^†^ (n = 6)	0.283 ± 0.114^†^ (n = 6)	3 ± 0.262^†^ (n = 6)	0.1 ± 2.539^†^ (n = 3)
	4 (361–700)	3.767 ± 0.478 (n = 3)	0.310 ± 0.106^†^ (n = 7)	0.310 ± 0.105^†^ (n = 7)	2.143 ± 0.243^†^ (n = 7)	0.1 ± 2.199^†^ (n = 4)
	5 (>700)	4.3 ± 0.828 (n = 1)	0.383 ± 0.162^†^ (n = 3)	0.383 ± 0.161^†^ (n = 3)	1.667 ± 0.371 (n = 3)	3.717 ± 2.539 (n = 3)^a^
Periumbilical	0	4.317 ± 0.338 (n = 6)	0.753 ± 0.114 (n = 6)	0.753 ± 0.114 (n = 6)	1 ± 0.262 (n = 6)	9.8 ± 2.199 (n = 4)
	1 (1–60)	4.008 ± 0.239 (n = 12)	0.624 ± 0.08 (n = 12)	0.624 ± 0.08 (n = 12)	1.667 ± 0.185 (n = 12)	0.14 ± 1.2^†^ (n = 9)
	2 (61–180)	4.396 ± 0.221 (n = 14)	0.344 ± 0.07^†^ (n = 14)	0.374 ± 0.074 (n = 14)	2.857 ± 0.172^†^ (n = 14)	0.117 ± 1.391^†^ (n = 10)
	3 (181–360)	4.933 ± 0.338 (n = 6)	0.268 ± 0.114^†^ (n = 6)	0.277 ± 0.114^†^ (n = 6)	3 ± 0.243^†^ (n = 7)	0.132 ± 3.11^†^ (n = 2)
	4 (361–700)	4.6 ± 0.586 (n = 2)	0.428 ± 0.14 (n = 4)	0.428 ± 0.14 (n = 4)	2.429 ± 0.243^†^ (n = 7)	3.912 ± 2.199^†^ (n = 4)
	5 (>700)	4.4 ± 0.414 (n = 4)	0.536 ± 0.125 (n = 5)	0.536 ± 0.124 (n = 5)	1.333 ± 0.262 (n = 6)	14.435 ± 2.199 (n = 4)^b^

* 1 = present; 2 = regressing/re-growing; 3 = absent

† Significant difference (*P* <0.05) between the observed value and initial value (time 0)

a Significantly different (*P* <0.05) from ^b^ between implant placement

All cats were in good health during the study and none showed any treatment-related side effects. Cat weight, post-GnRH serum testosterone concentrations, testicular size, presence of penile spikes, semen quality and reproductive behaviour during the study are shown in Tables 1–3. In 1/16 cats (cat 13) penile spikes never fully disappeared and testosterone always responded normally at every GnRH test, despite reproductive behaviour not being displayed for >1 year PT (400 days); therefore, this cat was considered as a non-respondent and its data were not included in the statistical analysis. There was no significant variation in body weight throughout the study and between the two groups of cats.

### Semen quality

Interval from treatment to complete sterility has been reported separately; sterility was complete at 40–72 days PT.^16^ Semen quality at the end of the study was documented in five cats (Tables 1 and 2): four of these cats (including cat 13 [the non-respondent]) had normal semen parameters with an interval between treatment and normal semen quality of 750–943 days (cats 2, 6 and 10), whereas one (cat 7) was azoospermic (despite the presence of penile spikes and normal serum testosterone).

### Serum testosterone

The linear regression formula of linearity test between observed testosterone values (ng/ml) and 1/dilution factor was y = 9.4973x + 0.7116 with an R² of 0.9888. The relationship between observed and expected values of the recovery test is expressed by the regression model y = 1.0777 × –0.3314 and the regression coefficient R^2^ was 0.9824. The coefficient of variation of every repeated assay was <15%. Our data on serum testosterone show good linearity, despite the slight overestimation of our chemiluminsescence immunoassay, which may be due to a matrix effect. Such overestimation may cause interpretative problems with values slightly above 1.0 ng/ml; however, the lowest value we had was 1.7 ng/ml.

**Figure 2:**
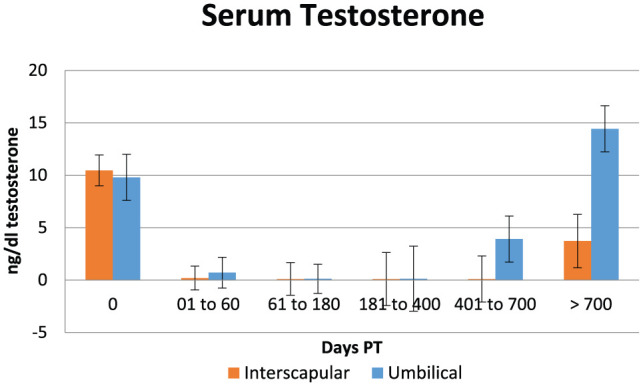
Graphical representation of mean (± SEM) serum testosterone concentration in 15 male cats treated with a 9.4 mg deslorelin implant between the shoulder blades (interscapular, orange columns) or 1.0 cm cranial to the umbilicus (umbilical, blue columns) from the day of treatment (day 0) until resumption of gonadal activity

Data on serum testosterone are shown in Tables 1–3 and Figure 2. Serum testosterone production decreased significantly between time periods 0 (treatment day: 9–11 ng/ml) and 1 (1–60 days PT: 0.14–0.18 ng/ml) (*P* <0.001) following both implant placements. Serum testosterone was already significantly decreased at 0.1–0.9 ng/ml in the four cats sampled 1 week PT. Serum testosterone in period 5 (>700 days PT) was significantly higher (3–14 ng/ml) than during periods 2–4, and did not differ from period 0 in both groups of cats. Resumption of fertility based on testosterone concentration rising to >1.0 ng/ml and the presence of normal penile spikes was observed between 750 and 850 days (Tables 1 and 2).

### Testicular volume

**Figure 3:**
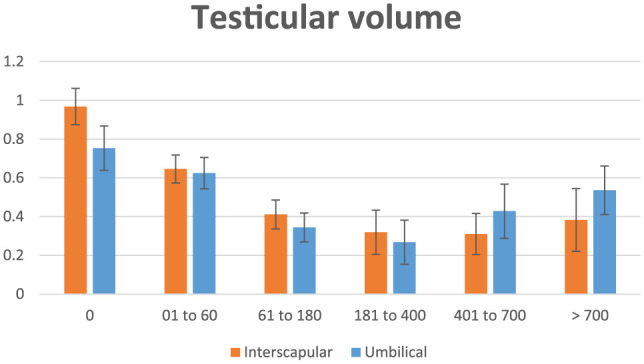
Graphical representation of testicular volume (cm^3^) in 15 male cats treated with a 9.4 mg deslorelin implant between the shoulder blades (interscapular, orange columns) or 1.0 cm cranial to the umbilicus (umbilical, blue columns) from the day of treatment (day 0) until resumption of gonadal activity

Data on testicular volume are shown in Tables 1–3 and Figure 3. No difference was found between implant location. However, there was a difference at some time periods in both right and left testicles (*P* <0.001): within the interscapular location, testicular volume was larger in time periods 0 and 1 vs time periods 2, 3, 4 and 5 for both right and left testicles (*P* <0.05); for periumbilical location, right testicular volume was larger in time periods 0, 1, 2, 4 and 5, and smaller only in time period 3 (*P* <0.05), whereas left testicular volume was larger in time periods 0, 1, 4 and 5 and smaller in time periods 2 and 3 (*P* <0.05), suggesting a delayed treatment effect following implant administration in the periumbilical area. Also, at the end of the study testicular volume started to increase earlier in cats of the periumbilical group (during time period 4, which is not statistically different from period 1, *P* = 0.47) as opposed to cats of the interscapular group (during period 5), suggesting a longer efficacy following implant administration in the interscapular area.

### Penile spikes

**Figure 4:**
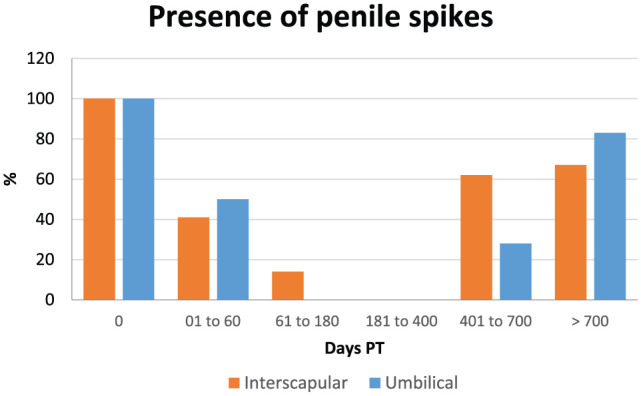
Graphical representation of the percentage of 15 male cats that had penile spikes present when treated with a 9.4 mg deslorelin implant between the shoulder blades (interscapular, orange columns) or 1.0 cm cranial to the umbilicus (umbilical, blue columns) from the day of treatment (day 0) until resumption of gonadal activity

Data on penile spikes are shown in Tables 1–3 and Figure 4. Penile spikes disappeared earlier (1–60 days PT) in cats implanted in between the shoulder blades (time period 0 different from time periods 1–4), than in cats implanted in the periumbilical area (61–180 days PT; time periods 0 and 1 different from time periods 2–4) (*P* <0.001). Penile spikes reappeared after a similar length of time (during time period 5 = >700 days PT) in both groups at the end of treatment effect.

### Histology

**Figure 5:**
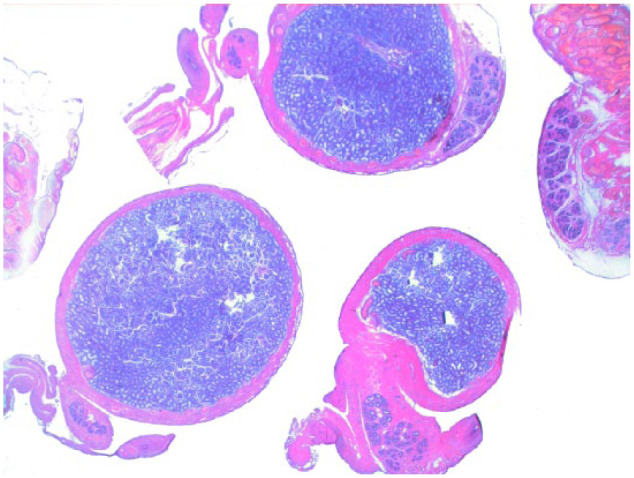
Cross section of the testicle of cat 14, which was treated with a 9.4 mg deslorelin implant and orchiectomised when still under the effect of treatment. Testis is smaller than normal, with thicker capsule and small seminiferous tubules and epididymis (haematoxylin and eosin, × 2 magnification)

**Figure 6:**
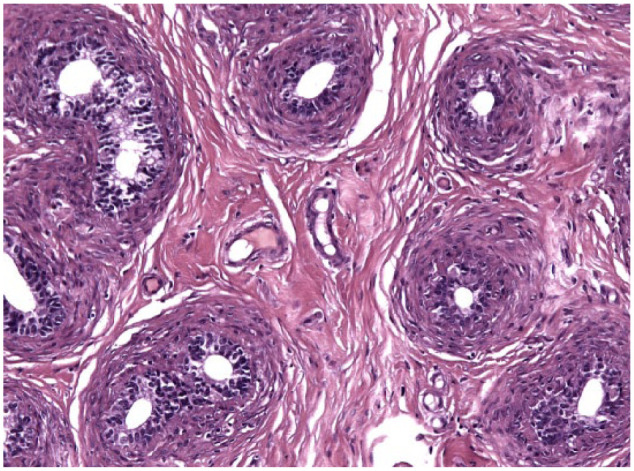
Epididymis of cat 14, which was treated with a 9.4 mg deslorelin implant and orchiectomised when still under the effect of deslorelin. Transverse section of small tubules lined by cuboidal cells with scant cytoplasm. Note moderate, dense collagenous stroma (haematoxylin and eosin, × 20 magnification)

**Figure 7:**
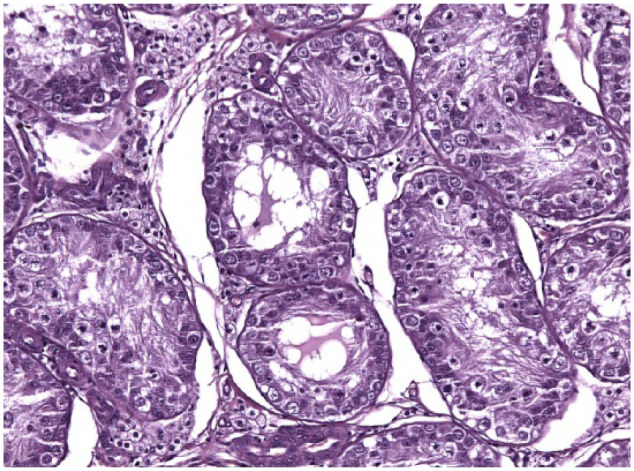
Testis of cat 14, which was treated with a 9.4 mg deslorelin implant and orchiectomised when still under the effect of deslorelin. Seminiferous tubules contain only large undifferentiated cells. Aggregates of small Leydig cells with poorly vacuolated cytoplasm are present between the tubules (haematoxylin and eosin, × 20 magnification)

**Figure 8:**
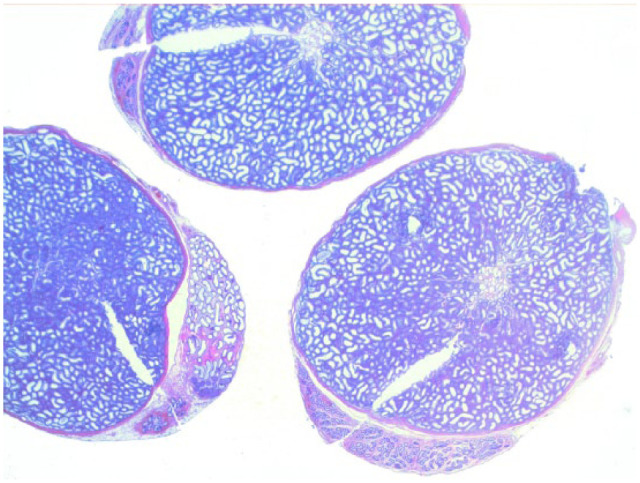
Testis of cat 10, which was treated with a 9.4 mg deslorelin implant and orchiectomised at the end of treatment following resumption of fertility. Serial sections of the testis show normal size of the organ with large seminiferous tubules, wide epididymis and a thin capsule (haematoxylin and eosin, × 20 magnification)

**Figure 9:**
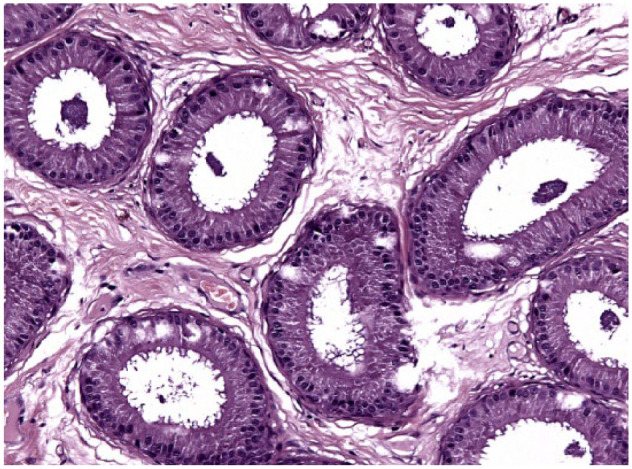
Epididymis of cat 10, which was treated with a 9.4 mg deslorelin implant and orchiectomised at the end of treatment following resumption of fertility. Transverse section of large tubules lined by cylindrical cells with abundant cytoplasm. Note loosely fibrillar stroma (haematoxylin and eosin, × 20 magnification)

**Figure 10:**
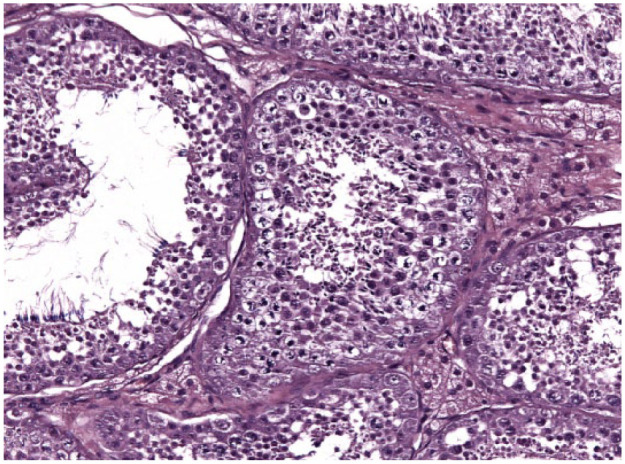
Testis of cat 10, which was treated with a 9.4 mg deslorelin implant and orchiectomised at the end of treatment following resumption of fertility. In the seminiferous tubules there are spermatogones visible close to the basal membrane, whereas spermatids, spermatocytes and spermatozoa are found within the wall of the seminiferous tubules and towards the lumen. Aggregates of hypertrophic Leydig cells with vacuolated cytoplasm are observed in between the tubules (haematoxylin and eosin, × 20 magnification)

Testicular histology was performed on 7/10 cats completing the study; for three cats (cats 6, 7 and 9) neutering was not requested by the owner. Two of seven cats (cats 12 and 14) were neutered while under treatment because of the early appearance of reproductive behaviour between days 120 and 200 (Tables 1 and 2), even though their serum testosterone was <0.1 ng/ml and penile spikes were absent. The other five cats (cats 2, 10, 11, 13 and 15) were neutered following their return to fertility (Tables 1 and 2). The two cats castrated during treatment showed lack of spermatocyte, mature spermatids or sperm in the seminiferous tubules (Figure 5); Leydig and Sertoli cells were atrophic, with small cytoplasmic vacuoles and hyperchromatic nuclei; epididymides were smaller than normal and coated with cubic cells with scarce-to-moderate pale cytoplasm (Figures 6 and 7). In the cats castrated following the end of the study, seminiferous tubules were lined by spermatids, spermatocytes and spermatozoa, and epididymides had cylindrical cells with abundant eosinophilic cytoplasm (Figures 8 and 9). Leydig and Sertoli cells were normal (Figure 10).

## Discussion

This is the first study in cats to report on: (1) onset and duration of reproductive control and resumption of fertility following treatment with a 9.4 mg deslorelin implant; (2) the use of the periumbilical area for implant placement; and (3) a validation protocol on feline serum testosterone assay using chemiluminescence. No skin reactions in the two different implant locations were observed. In cats, injection site sarcomas have been reported at 6 weeks after the injection.^22^ Pathogenesis of this condition is still not clear. Injection site sarcoma can occur after injection of a variety of medications. The administration of long-acting penicillin and corticosteroids, lufenuron, as well as microchip insertion or vaccination, have been associated with development of sarcomas in cats.^23^ The cats of our study were observed for up to 3 years and no abnormality was observed at the site of implant administration.

On average, intervals between treatment and resumption of normal serum testosterone concentrations with the presence of penile spikes was 805 days (range 750–850 days; n = 4) and between treatment and resumption of semen production it was 835 days (range 750–943 days; n = 3). The marked difference in duration of reproductive control in cats when compared with the 4.7 mg deslorelin implant makes the 9.4 mg implant very attractive for clinical use both in client-owned cats and in stray and feral cats (for which life expectancy is far shorter than pet cats). A prolonged duration of the 9.4 mg deslorelin implant has also been reported in dogs (up to 400 days),^24,25^ snakes (>700 days) and in wild hogs (700–1000 days).^26^

Semen quality showed an initial improvement, despite the concomitant fall of serum testosterone to basal values: this may reflect a stimulation of sperm maturation due to a peak in serum testosterone presumably occurring 2–4 days PT as reported.^3,9,16,27^ Semen quality started to decrease during the second month PT and by 72 days all cats were diagnosed as sterile.^16^ The fact that one of these four cats was azoospermic when collected at the end of the study is difficult to explain. A reduced pituitary (low luteinising hormone and follicle-stimulating hormone release) and testicular (low Leydig cell function) responsiveness could play a role in cats similar to what has been hypothesised as a cause for azoospermia in male dogs treated with deslorelin.^3^ However, this cat had the longest interval (72 days) between treatment and sterility;^18^ considering biological variability, it is possible that his semen production may have taken longer to be re-established.

Serum testosterone remained at basal levels for a similar duration of time and returned to normal levels beyond 700 days PT in both groups. However, at the end of treatment (>700 days; n = 7) average serum testosterone concentration was lower in cats implanted between the shoulder blades (3.7 ng/ml) than in cats implanted in the periumbilical area (14.4 ng/ml; *P* <0.05). Although a post-GnRH serum testosterone value of 3.7 ng/ml is compatible with normal reproductive function, this result may indicate that full testicular activity is reached sooner in cats implanted in the periumbilical area.

In 15/16 cats in which treatment was successful, testicular volume decreased by 24% at 2 months, 50% at 6 months and 60% at 12 months. In both locations the maximum treatment effect (lack of penile spikes and smaller testicular volume) was observed during time period 3 (181–360 days PT). Towards the end of the study (time period 4 = 361–700 days PT) testicular volume was still significantly lower than in time period 0 in cats of the interscapular group, whereas it did not differ any longer from time period 0 in cats of the periumbilical group (Table 3), suggesting a longer lasting effect of deslorelin when administered in between the shoulder blades. However, the fact that there was no difference in testicular volume between time period 5 in cats of both groups (Table 3) shows that total duration of effect was the same, although the action of deslorelin may be stronger causing a deeper inhibition of testicular function in cats implanted in the interscapular area.

Such a difference in the magnitude of action of a deslorelin implant placed in the interscapular area is difficult to explain. Deslorelin treatment has also been associated with increased appetite and weight gain in cats.^9,26^ Accumulation of fat, which occurs more commonly in the abdominal area, might affect the speed with which the drug is metabolised in animals implanted in the periumbilical area. The reason why left and right testicular volume decreases at a different pace in cats implanted in the periumbilical area is also difficult to explain, as it could be due to small sample size or the fact that not all measurements could be performed independently by two operators.

Penile spikes began to regress at 8 ± 4 weeks and disappeared at 14 ± 5 weeks, similarly to what has been previously observed using the 4.7 mg deslorelin implant.^10,15^ Regression started earlier in cats implanted in the interscapular area. Collectively, serum testosterone, testicular volume and penile spike data seem to indicate that the interscapular location was characterised by a more efficacious (faster onset and stronger action) effect of the 9.4 mg deslorelin implant in the cats of our study. However, because of the relatively small number of animals in our study and the fact that not all clinical observations could be made independently by two operators, further studies are needed before a definitive conclusion on this issue can be made.

Reproductive behaviour resumed earlier than semen output and serum testosterone secretion: of 11 cats for which we have extended monitoring, three showed roaming and/or mounting activity at 120 (cat 14), 200 (cat 12) and 660 (cat 10) days PT without penile spikes and in the presence of basal serum testosterone concentrations (Tables 1 and 2). Cats 12 and 14 may have come into contact with oestrous queens, whereas cat 10 was living indoors under constant supervision and without any contact with females. The occurrence of patterns typical of intact males in these cats may be due to learned behaviour not being affected by deslorelin.

## Conclusions

Treatment with a 9.4 mg deslorelin implant in male cats in this study was effective for a period of 750–850 days, which is 1.5–2 times longer than the effect of the 4.7 mg deslorelin implant.^9–12,15,28^ The two different placements (intrascapular vs periumbilical) did not affect duration of suppression of testosterone production. The interscapular location may be characterised by a better efficacy, although further studies are needed to clarify this issue.

Failure to respond to deslorelin in cat 13 is difficult to explain. The presence of the implant in the subucutaneous tissue of this cat could not be assessed over time as palpation of implants placed in the interscapular area is impossible. Implant insertion in this cat was uneventful, penile spikes showed a partial decrease and an effect on behaviour was evident until 430 days PT. Semen collection performed at time of castration (550 days PT) showed normal semen parameters. Although semen collection could not be performed during the study in this cat, normal semen quality is likely to have been present considering the normal response to the GnRH stimulation test.

The occasional occurrence of a non-responding cat has also been reported following the use of the 4.7 mg implant.^12^ In addition, failure to respond to deslorelin has been reported in monkeys (white-faced saki), raccoons (brown-nosed coati) and lionesses (*Panthera leo*).^26^ Although highly effective in cats, deslorelin may not always reach 100% efficacy in tom cats, unlike what has been found in queens.^12,29–31^ At the end of the study normal testicular function (based on secretion of serum testosterone, presence of penile spikes, normal semen production either through semen collection or testicular histology) was re-established.
